# Comparison between Two Different Scanners for the Evaluation of the Role of ^18^F-FDG PET/CT Semiquantitative Parameters and Radiomics Features in the Prediction of Final Diagnosis of Thyroid Incidentalomas

**DOI:** 10.3390/jcm11030615

**Published:** 2022-01-26

**Authors:** Francesco Dondi, Nadia Pasinetti, Roberto Gatta, Domenico Albano, Raffaele Giubbini, Francesco Bertagna

**Affiliations:** 1 Nuclear Medicine, Università degli Studi di Brescia and ASST Spedali Civili Brescia, 25123 Brescia, Italy; f.dondi@outlook.com (F.D.); raffaele.giubbini@unibs.it (R.G.); francesco.bertagna@unibs.it (F.B.); 2 Radiation Oncology Department, ASST Valcamonica Esine and Università degli Studi di Brescia, 25040 Brescia, Italy; nadia.pasinetti@unibs.it; 3 Dipartimento di Scienze Cliniche e Sperimentali dell’Università degli Studi di Brescia, 25123 Brescia, Italy; roberto.gatta@unibs.it

**Keywords:** thyroid incidentalomas, radiomics, texture analysis, ^18^F-FDG, PET/CT, positron emission tomography, thyroid cancer

## Abstract

The aim of this study was to compare two different tomographs for the evaluation of the role of semiquantitative PET/CT parameters and radiomics features (RF) in the prediction of thyroid incidentalomas (TIs) at ^18^F-FDG imaging. A total of 221 patients with the presence of TIs were retrospectively included. After volumetric segmentation of each TI, semiquantitative parameters and RF were extracted. All of the features were tested for significant differences between the two PET scanners. The performances of all of the features in predicting the nature of TIs were analyzed by testing three classes of final logistic regression predictive models, one for each tomograph and one with both scanners together. Some RF resulted significantly different between the two scanners. PET/CT semiquantitative parameters were not able to predict the final diagnosis of TIs while GLCM-related RF (in particular GLCM entropy_log2 e GLCM entropy_log10) together with some GLRLM-related and GLZLM-related features presented the best predictive performances. In particular, GLCM entropy_log2, GLCM entropy_log10, GLZLM SZHGE, GLRLM HGRE and GLRLM HGZE resulted the RF with best performances. Our study enabled the selection of some RF able to predict the final nature of TIs discovered at ^18^F-FDG PET/CT imaging. Classic semiquantitative and volumetric PET/CT parameters did not reveal these abilities. Furthermore, a good overlap in the extraction of RF between the two scanners was underlined.

## 1. Introduction

Differentiated thyroid cancer (DTC) represents about 1% of all malignant tumors; moreover, it is the most frequent form of endocrine carcinoma and is usually characterized by good prognosis [1,2,3,4]. In recent years, its incidence has been growing due to the increasing use of needle aspiration and thyroid ultrasound [5,6,7].

The role of nuclear medicine in the diagnostic and therapeutic work-up of DTC is pivotal. In fact, nowadays, exams performed with 131I are fundamental for the staging, the restaging, and the therapy of this carcinoma [4,8].

In recent years, we have been continuously experiencing an increase in the use of positron emission tomography/computed tomography (PET/CT) with ^18^F-fluorodeoxyglucose (^18^F-FDG) for the evaluation of various pathologies, both neoplastic and inflammatory. In this context, even in the diagnostic work-up of DTC this hybrid imaging modality has a central role, in particular in the evaluation of patients with no evidence of 131I avid disease but a persistence of elevated thyroglobulin levels [4,9,10].

With the increasing use of ^18^F-FDG PET/CT in the clinical practice, we have also been experiencing an increase in the detection of thyroid incidentalomas (TI) [11,12,13]. TIs are defined as thyroid lesions detected at imaging studies performed for non-thyroid pathologies [14,15].

The precise evaluation of TIs is mandatory, given the non-negligible risk of presence of CTD in a high amount of these findings [16,17,18]. In this context, a lot of authors have tried to clarify the role of ^18^F-FDG PET/CT for the definition of the precise nature of TIs, in terms of malignancy or benignancy [4]. However, the role of some PET/CT semiquantitative parameters, such as standardized uptake value (SUV), metabolic tumor volume (MTV) and total lesion glycolysis (TLG) has not yet been fully clarified and the results in literature are really heterogeneous [4].

Furthermore, in recent years we have been appreciating an increase in the extraction of specific quantitative features from PET images, called radiomics or texture analysis. In this setting, the use of radiomics for the correct evaluation of every type of incidentalomas is waking increasing interest [19,20]. The case of TIs is not an exception and some works about the use of texture analysis for their correct classification have been produced [21,22,23,24]. However, similarly to semiquantitative parameters, the use of radiomics in this setting has given non-clarifying and initial results.

The aim of this retrospective study is to evaluate the role of semiquantitative PET/CT parameters and radiomics features for the correct classification of TIs discovered at ^18^F-FDG PET/CT scans. Furthermore, the impact of different PET/CT tomographs on texture analysis and on its ability to predict the final outcome is a fundamental part of this work.

## 2. Materials and Methods

### 2.1. Patients Selection

We retrospectively analyzed the ^18^F-FDG PET/CT scans performed in our center between January 2012 and December 2020 in order to find presence of TIs. All of the patients performed PET/CT exams for staging or restaging purpose of various diseases, but no one had a previous history of DTC. Specifically, 82 patients suffered from lymphoma, 19 from carcinomas of the head and neck, 51 from lung cancer, 6 from fever of unknown origin, 12 from vasculitis, 38 from breast cancer, 3 from esophageal cancer, 2 from ovarian cancer, 5 from colorectal cancer and 2 from endocarditis, while 1 patient performed the examination in order to characterize a formation of the right adrenal gland.

Tis were defined as focal uptakes of ^18^F-FDG inside the thyroid gland with an uptake higher than the background uptakes. Given the fact that diffuse uptakes on thyroid gland are usually expression of benign conditions, they were excluded from the study [4]. Furthermore, other inclusion criteria were the presence of an ultrasound follow-up of at least 1 year for suspected benignant uptakes and the execution of a cytological evaluation and/or histological examination for suspected malignant uptakes. A total of 237 patients were therefore included in the study and data about the lobe of TIs and ultrasound dimension were collected.

### 2.2. ^18^F-FDG PET/CT Acquisition and Interpretation

^18^F-FDG PET/CT scans were acquired after at least 6 h of fasting and with blood glucose levels below 150 mg/dL. An activity of 3.5–4.5 MBq/kg of ^18^F-FDG was intravenously administrated to the patients 1 hour before images acquisition. Images were acquired from the base of the skull to the mid-thigh. All of the patients were instructed to void before the PET/CT acquisition and no type of oral or intravenous contrast agents were given for the execution of the scan. Similarly, none of the patients had performed any intestinal preparation.

In our study, we made use of 2 different PET/CT tomographs: the first (scanner 1) was a Discovery 690 PET/CT (General Electric Company-Milwaukee, WI, USA) while the second (scanner 2) was a Discovery STE PET/CT (General Electric Company, Milwaukee, WI, USA). On both of them standard acquisition parameters (CT: 80 mA, 120 Kv without contrast; 2.5–4 min per bed- PET-step, axial width 15 cm) and standard reconstruction parameters were used (256 × 256 matrix and 60 cm field of view).

Furthermore, scanner 1 was characterized by the presence of LYSO (cerium-doped lutetium yttrium oxyorthosilicate) scintillator crystals with a decay time of 45 ns, while scanner 2 had BGO (bismuth germanate) scintillator crystals with a decay time of 300 ns. Scanners were not harmonized with a cross-calibration program and all PET/CT scans were acquired at free-breath, instructing the patients to have regular breathing. For both scanners, a low dose CT at free breathing and without contrast agent was acquired in order to perform attenuation correction and for anatomical correlation. In particular, CT acquisition parameters for scanner 1 were: 120 kV, fixed tube current ≈ 60 mAs (40–100 mAs), 64 slices × 3.75 mm and 3.27 mm interval, pitch 0.984:1, tube rotation 0.5 s. CT acquisition parameters for scanner 2 were: 120 kV, fixed tube current ≈ 73 mAs (40–160 mAs), 4 slices × 3.75 mm and 3.27 mm interval, pitch 1.5:1, tube rotation 0.8 s. Furthermore, on scanner 1 time of flight (TOF) and point spread function (PSF) algorithm were used for the reconstruction of images, with filter cut-off 5 mm, 18 subsets and 3 iterations. Again, on scanner 2, an ordered subset expectation maximization (OSEM) algorithm with filter cut-off 5 mm, 21 subsets and 2 iterations were used.

PET images were visually and semiquantitatively analyzed by a nuclear physician with at least 10 years of experience, measuring parameters of TIs: the maximum standardized uptake value corrected for body weight (SUVmax), mean SUV corrected for body weight (SUVmean), maximum standardized uptake value lean body mass (SUVlbm), maximum standardized uptake value body surface area (SUVbsa), MTV and TLG. SUV-related parameters were measured on a Xeleris 3.1 GE workstation. MTV was calculated by drawing a volume of interest (VOI) on TIs on ^18^F-FDG PET/CT images corrected for attenuation, using a SUV-based automated contouring program (Advantage Workstation 4.6, GE HealthCare) with an isocounter threshold method based on 41% of the SUVmax, as previously recommended by the European Association of Nuclear Medicine (EANM) because of its high inter-observer reproducibility [25]. TLG values were calculated as the product of the MTV of the VOI for its SUVmean.

### 2.3. Radiomics Features Extraction

Image features were extracted from PET images by using LIFEx 2.20 software (LIFEx, by the French Alternative Energies and Atomic Energy Commission (CEA), Gif-sur-Yvette, France) (http://www.lifexsoft.org, accessed on 10 September 2021) [26] with the same procedure previously described for SUV-related parameters extraction, with similar VOI and after a new segmentation process.

The extraction of radiomics features (RF) was performed without spatial resampling, with an intensity discretization of 64 grey levels and with a distance from neighbors of 1 voxel for the extraction of GLCM parameters.

A total of 42 RF were generated (Table 1), divided in first-order statistics (histogram-related and shape-related) and second-order statistics: grey level co-occurrence matrix (GLCM) related, grey-level run length matrix (GLRLM) related, neighborhood grey level different matrix (NGLDM) related and grey-level zone length matrix (GLZLM) related.

### 2.4. Statistical Analysis

Statistical analysis was performed using MedCalc Software version 18.1 (8400, Ostend, Belgium) and R (http://www.R-project.org/) software version 4.1.1 (Statistics Department of the University of Auckland, Auckland, New Zealand). In the descriptive analysis, the categorical variables were represented as simple and relative frequencies, while the numeric variables with mean, standard deviation, and range values. For both scanners, the kernel density estimation built on the RF values were qualitatively compared and the presence of significant differences were evaluated with the Mann–Whitney test.

The general statistical analysis line of the study was structured of various steps. First of all, a univariate analysis (with a logistic regressor, in a 10-cross-fold validation) was performed for the group of patients evaluated on scanner 1, 1 for the group of patients of scanner 2 and 1 for the entire group of patients (scanner 1 and scanner 2 considered together). This first analysis had the purpose to evaluate the influence of the two scanners on the ability of RF to correlate with the final clinical outcome.

Furthermore, a bivariate analysis was performed with the purpose of developing 3 predictive models (1 for scanner 1, 1 for scanner 2 and 1 for both scanners considered together), by analyzing all of the possible couples of variables (the cartesian product of semiquantitative parameters, RF and the major clinical features such as age, gender and ultrasound dimension of the Tis). This bivariate analysis was performed with a bivariate logistic regression model was applied in order to classify them on the basis of the area under the curve (AUC) under the receiving operator curve (ROC) after a 10-cross fold validation training/testing test. This bivariate model had the purpose to clearly explore all the space of RF presented in the study. Similarly, for each couple of variables, the accuracy was extrapolated and to obtain a more complete statistic, the *p*-value were also extracted.

Lastly, a selection of the models with the best bivariate logistic regression was performed for scanner 1, scanner 2 and for both scanners considered together. In this setting an AUC higher than 0.8 was arbitrarily considered optimal to predict the final diagnosis of TIs, while an AUC between 0.6 and 0.8 was considered acceptable. Similarly, a *p*-value < 0.05 was arbitrarily considered as statistically significant.

## 3. Results

### 3.1. Patients Characteristics

A total of 221 patients were included in the study (Table 2), with a mean age of 66 years (range 16–88). The majority of the patients were female (*n* = 149, 67%) while 72 (33%) were male. No significant difference in terms of sex between the 2 groups of malignant TIs and benignant TIs was underlined (*p* value = 0.07).

TIs were most frequent findings on the right thyroid lobe with 123 (56%) subjects, while in 87 (39%) the incidental uptake were discovered on the left lobe and only in 11 (5%) cases they were underlined at the isthmus. Again, the site of TIs was not significantly correlated with the final diagnosis (*p* value = 0.79).

The mean diameter of the TIs, evaluated on subsequent ultrasound evaluation, was of 17 mm (range 5–75).

Overall, the final diagnosis of TIs was malignant for 71 (32%) patients and benignant in 150 (68%) patients. In this setting, for the correct evaluation of their final diagnosis, 97 (44%) subjects were evaluated only with ultrasound exams, with a mean follow-up of 24 months (range 12–168). Five (2%) patients performed a ^99m^Tc thyroid scintigraphy, that revealed the presence of an hyperfunctioning adenoma.

For 118 (53%) patients, a cytological examination for the correct diagnosis of incidental ^18^F-FDG uptakes was performed, classifying the results according to the Italian Thyroid Cytology Classification System [27]. In particular, in 16 (14%) cases the result of the cytological examination was TIR5, in 13 (11%) it was TIR4, in 54 (45%) it was TIR3 while in 35 (30%) it was TIR2. Furthermore, of the 54 patients with a TIR3 classification, 24 (44%) had a TIR3a result while TIR3b was the final cytological result for 30 (56%) patients. A histological diagnosis of the TIs was performed in 71 (32%) cases and all of them revealed the presence of malignancy. In particular, in 3 (4%) cases the presence of anaplastic carcinoma was revealed, in 7 (10%) the presence of follicular carcinoma was underlined and in 61 (86%) there was a final diagnosis of papillary carcinoma. An evaluation of the predictive abilities of semiquantitative PET/CT parameters and of RF to predict the final cytological or histologic diagnosis was not performed because of the low sample of subjects beneath all the subgroups mentioned before.

**Figure 1:**
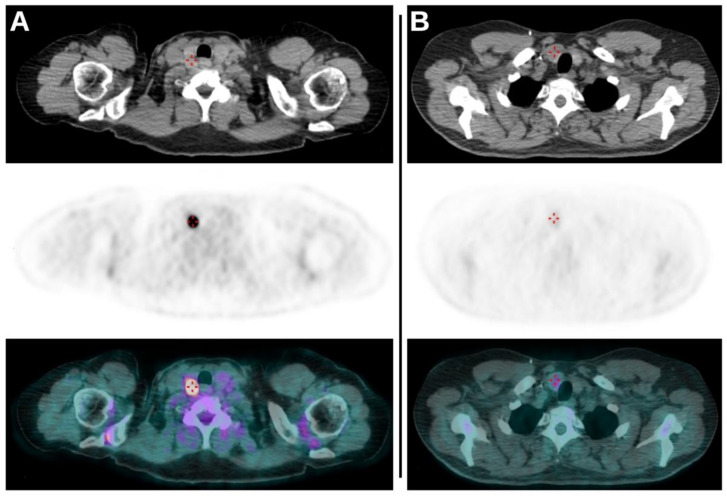
(**A**): Axial CT, axial PET and axial fused PET/CT images demonstrating the presence of TI revealed as intense focal uptake of ^18^F-FDG on the right lobe of thyroid. The lesion had a SUVmax of 44.47, an MTV of 0.7 and a TLG of 18.1 and subsequent cytological exam revealed no malignancy (TIR2). (**B**): Axial CT, axial PET and axial fused PET/CT images of another scan demonstrating again the presence of TI as a faint uptake on the right lobe of thyroid. The values of SUVmax, MTV and TLG of the lesion were 2.64, 6.9 and 10.3, respectively. Cytological evaluation (TIR5) and subsequent total thyroidectomy revealed the presence of papillary carcinoma.

A total of 128 (58%) scans were performed on the Discovery 690 tomograph (scanner 1), while 93 (42%) of them were acquired on the Discovery STE tomograph (scanner 2). The mean value of the SUVmax of the TIs was 7.9, it was 4.3 for SUVmean, 5.8 for SUVlbm, 2.0 for SUVbsa, 9.2 for MTV and 35.0 for TLG. (Figure 1).

Analyzing PET/CT acquisition depending on the tomograph used for their execution, in 92 (72%) scans performed on scanner 1 the incidental uptake resulted of benign nature while in 36 (28%) cases the final diagnosis was malignancy (1 anaplastic carcinoma, 4 follicular carcinomas and 31 papillary carcinomas). Regarding scanner 2, in 58 (62%) scans the final diagnosis of incidental uptake was benignancy while in 35 (38%) cases the presence of malignancy was underlined (2 anaplastic carcinomas, 3 follicular carcinomas and 30 papillary carcinomas). No significant difference in terms of final diagnosis was reported between the 2 scanners (*p* value = 0.1).

### 3.2. Comparison between the Two Scanners

The major clinical and epidemiological characteristics of the patients (age, sex, ultrasound dimension and final diagnosis of the TIs) were not significantly different between the two scanners.

Regarding semiquantitative parameters of PET/CT, only the values of SUVmax resulted significantly different between the 2 scanners (*p* value = 0.046), while the remaining parameters were not. In particular, the SUVmax values resulted higher on scanner 1 compared to scanner 2.

**Figure 2:**
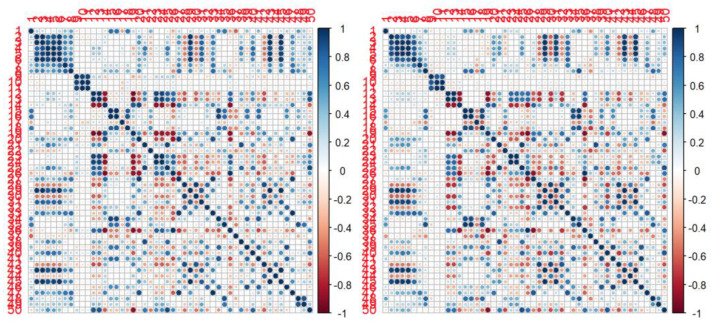
Correlation maps for first and second order RF between the two scanners. Scanner 1 (Discovery 690) is presented on the left, while scanner 2 (Discovery STE) is presented on the right. Blue means high positive correlation; red means high negative correlation; white means no correlation.

Focusing on RF, only 9 of 42 resulted in significant differences between the 2 scanners. In particular, RF with apparent correlation on the type of scanner used for the acquisition were Histo entropy_log10, Histo entropy_log2, Histo Energy, GLRLM LGRE, GLRLM SRLGE, NGLDM busyness, GLZLM SZE, GLZLM SZHGE and GLZLM ZLNU (Table 3). However, cross-correlation maps of RF between the two scanners were quite similar (Figure 2).

### 3.3. Predictive Accuracy

At univariate analysis (Table 4), for scanner 1 (Discovery 690) all PET/CT semiquantitative parameters and RF obtained an AUC value between 0.6 and 0.8. Regarding scanner 2 (Discovery STE), again all of the semiquantitative PET/CT parameters and RF reached a value of AUC between 0.6 and 0.8; in general, these values were lower than the those reported for scanner 1. Furthermore, the evaluation of *p*-value allowed the selection of some parameters with the best performances for the prediction of the final diagnosis of Tis, for both scanner 1 and scanner 2.

Considering the combined analysis of both the scanners together (scanner 1 + 2), in general PET/CT semiquantitative parameters revealed a higher AUC compared to RF, with significant *p*-value. Interestingly, the combined evaluation of both scanners revealed acceptable values of UAC with significant *p*-value for some RF, even if the same RF did not reach these values at the analysis for single scanner.

**Figure 3:**
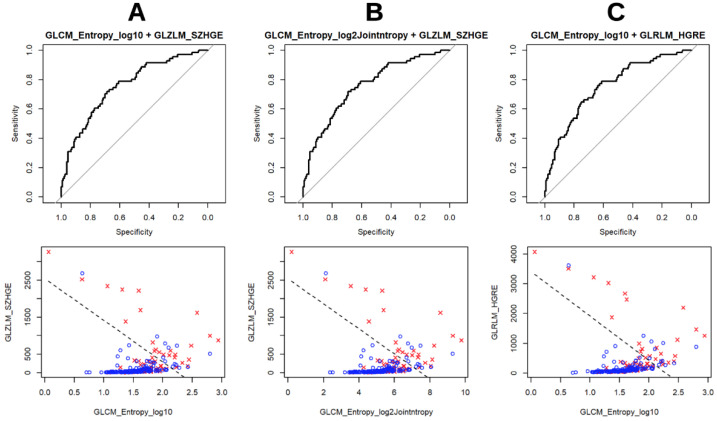
Visual representations of the three combinations ((**A**) GLCM Entropy_log10+GLZLM_SZHGE, (**B**) GLCM Entropy_log2+GLZLM:SZHGE; (**C**) GLCM Entropy_lo10+GLRLM_HGRE) with best performances at bivariate analysis for both scanners considered together.

After performing a bivariate analysis, for both the single scanners and for both of the scanners considered together, the best combinations between PET/CT semiquantitative parameters and RF are summarized in Table 5. Similarly to univariate analysis, none of the combinations reached an optimal AUC of 0.8 and the couples of parameters generally obtained higher AUC values on scanner 1 than on scanner 2. Furthermore, for this analysis, the p-values were statistically more significant on scanner 1 than on scanner 2. In this setting, even if a comparison between the couples of variables obtained before is complex given the heterogeneity between the two scanners, in general GLCM-related parameters variously combined resulted the ones with best performances. This is true for both scanner 1 and scanner 2 and these findings are confirmed by the good results at univariate analysis previously described. The GLRLM-related and GLZLM-related RF also revealed good performances in this setting. Interestingly, PET/CT semiquantitative parameters were confirmed as good predictors only for scanner 2 (Figure 3).

## 4. Discussion

The aim of this study was to verify the predictive abilities of semiquantitative PET/CT parameters and of RF to discriminate between benignant and malignant nature of TIs revealed at ^18^F-FDG imaging.

On the basis of the resulting evidence we identified some remarkable points concerning the effect of different PET scanners on RF extraction and the predictive features and associated models.

In our experimental setting, we had to deal with images coming from different PET/CT tomographs and this fact required a preliminary investigation of the effect of different technologies in producing images and subsequent image features. The results showed that the scanner technology concretely affects some RF, as previously underlined in literature, and in clinical day practice the use of different tomographs in the same department is frequent [20,28,29,30,31,32,33,34]. In particular, the acquisition of the same phantoms on different tomographs with different scintillators and algorithm used for the reconstruction (number of iterations, number of subsets or on the presence of partial volume correction) demonstrated this evidence.

These findings suggest two relevant points: the former indicates that different scanners can potentially have different preferred features in terms of correlations with a clinical outcome; the second point suggests that we must critically consider radiomics models coming from centers adopting different technologies. In other words, on one hand a unique radiomic best model trained on many scanners is probably suboptimal for each of them and on the other hand, any radiomic model coming from different centers should be internally validated before considering its use in the daily practice. In particular, in the literature only one study which evaluated the predictive role of RF in TIs [23] used different scanners for the extraction of RF: this means that the reproducibility of the results (which is one of the biggest challenges in radiomics) still remain uninvestigated in this field. Furthermore, in our evaluation, only a small amount of RF demonstrated to be significantly different between the two scanners, together with SUVmax, but nevertheless the cross-correlation maps resulted quite similar, adding value to our results. In this setting, of the parameters that after bivariate analysis demonstrated the best performances, GLZLM SZHGE was the only one significantly different between the two scanners.

Regarding the predictive role of RF for the correct evaluation of Tis, at univariate and bivariate analysis a good percentage of the aforementioned parameters revealed an acceptable AUC between 0.6 and 0.8. However, none of them demonstrated an AUC above 0.8. Similarly, these AUC were coupled with a significant *p*-value in a high percentage of the cases. It is worth underlining the fact that at bivariate analysis performed for both the scanner considered together, the AUC values and the *p-*values were the best in the whole study. This fact underlines a good predictive ability of some RF such as GLCM-related (in particular GLCM entropy_log2 e GLCM entropy_log10), GLRLM-related and GLZLM-related.

Only a small amount of works that investigate the predictive role of radiomics in the evaluation of TIs at ^18^F-FDG PET/CT are available in literature [21,22,23,24].

Even if not clearly characterized by the presence of a proper texture analysis, the first study to evaluate the distributive heterogeneity of ^18^F-FDG in TIs was produced by Kim et al. [24]. In this work, the authors revealed that this heterogeneity was a promising parameter which was able to predict the final nature of these TIs. 

Subsequently, Sollini et al. [23] were the first to evaluate the predictive abilities of texture analysis in this setting. Data of this study underlined the fact that SUVstd (the standard deviation of the distribution of SUV inside the considered VOI), SUVmax, MTV, TLG, Histo skewness, Histo kurtosis and GLCM correlation were the only parameters that were able to predict the final diagnosis of TIs, with a general positive predictive value of 54% and a general negative predictive value of 85%.

A similar analysis was also performed by Aksu et al. [22], who underlined how the semiquantitative PET/CT parameters and some shape-related, GLCM-related, GLRLM-related and GLZLM-related RF obtained AUC values superior to 0.7. These findings were partially confirmed in our study, where the same parameters confirmed these good results, with the exception of semiquantitative parameters and shape-related RF. Furthermore, the authors of the study developed a machine-learning algorithm using GLRLM RLNU e SUVmax with a good general AUC value (0.731).

Lastly, Ceriani et al. [21] demonstrated the ability to predict the final nature of TIs of some PET/CT semiquantitative parameters (SUVmax, SUVmean, SUVpeak, MTV e TLG) and some RF. In this case, the authors performed texture analysis with a different software from LIFEx and so RF resulted partly different in comparison to the ones used in our study. In general, some shape-related and GLCM-related features demonstrated good performances and multivariate analysis confirmed TLG, SUVmax and Shape sphericity as able to predict the final nature of Tis.

It is interesting to underline that PET/CT semiquantitative parameters resulted good predictors in all of the studies, while in our work only SUVmean obtained a certain predictive role at bivariate analysis. In this setting, we reported that AUC of semiquantitative parameters were quite similar to AUC of RF only at monovariate analysis. Given the fact that the bivariate predictive model did not confirm this evidence, we can assume that these parameters do not perform well when trying to build models with multiple variables as in our case. Furthermore, as previously described, data in literature about the role of these parameters for the assessment of TIs are really heterogeneous and our findings confirm these insights. Moreover, RF describe quality and parameters of images that cannot be visually assessed and this is why we focused our attention on the evaluation of these features, allowing us to better understand the role of ^18^F-FDG PET/CT in the prediction of Tis nature.

Our study surely presents some limitations. First of all, this is a retrospective study with the use of tomography that are not the actual state. Furthermore, the relatively low sample of patients included in the work, even if higher than similar studies, appears sub-optimal to clearly evaluate the predictive abilities of texture analysis. Furthermore, RF extrapolation with a single software appears another limit of our analysis. Lastly, the aforementioned problem of the reproducibility of radiomics analysis in terms of multicentric evaluation is still an open issue and, in this setting, further research in this field are mandatory.

## 5. Conclusions

In conclusion, our study enabled the selection of some RF that are able to predict with a certain good accuracy, the final nature of TIs discovered at ^18^F-FDG PET/CT imaging. Classic semiquantitative and volumetric PET/CT parameters did not reveal this ability. Furthermore, a good overlap in the extraction of RF between the two scanners was underlined.

## Figures and Tables

**Table 1:** List of semiquantitative parameters and of radiomics features considered in the study.

Semiquantitave Parameters
SUV-related
SUVmax
SUVmean
SUVlbm
SUVbsa
Volumetric parameters
MTV
TLG
**Radiomics features**
First order features
Histogram related
Histo skewness
Histo kurtosis
Histo excess kurtosis
Histo entropy_log10
Histo entropy_log2
Histo energy
Shape related
Shape volume_mL
Shape volume_vx
Shape sphericity
Shape compacity
Second order features
Grey level co-occurrence matrix (GLCM) related
GLCM homogeneity
GLCM energy
GLCM contrast
GLCM correlation
GLCM entropy_log10
GLCM entropy_log2
GLCM dissimilarity
Grey-level run length matrix (GLRLM) related
GLRLM SRE
GLRLM LRE
GLRLM LGRE
GLRLM HGRE
GLRLM SRLGE
GLRLM SRHGE
GLRLM LRLGE
GLRLM LRHGE
GLRLM GLNU
GLRLM RLNU
GLRLM RP
Neighborhood grey level different matrix (NGLDM) related
NGLDM coarseness
NGLDM contrast
NGLDM busyness
Grey-level zone length matrix (GLZLM) related
GLZLM SZE
GLZLM LZE
GLZLM LGZE
GLZLM HGZE
GLZLM SZLGE
GLZLM SZHGE
GLZLM LZLGE
GLZLM LZHGE
GLZLM GLNU
GLZLM ZLNU
GLZLM ZP

SUVmax: standardized uptake value body weight max; SUVmean: standardized uptake value body weight mean; SUVlbm: standardized uptake value lean body mass, SUVbsa: standardized uptake value body surface area; MTV: metabolic tumor volume; TLG: total lesion glicolysis; SRE: short-run emphasis; LRE: long-run emphasis; LGRE: Low Gray-level Run Emphasis; HGRE: High Gray-level Run Emphasis; SRLGE: Short-Run Low Gray-level Em-phasis; SRHGE: Short-Run High Gray-level Emphasis; LRLGE: Long-Run Low Gray-level Emphasis; LRHGE: Long-Run High Gray-level Emphasis; GLNU: Gray-Level Non-Uniformity; RLNU: Run Length Non-Uniformity; RP: Run Percentage; SZE: Short-zone emphasis; LZE: Long-zone emphasis; LGZE: Low Gray-level Zone Emphasis; HGZE: High Gray-level Zone Emphasis; SZLGE: Short-Zone Low Gray-level Emphasis; SZHGE: Short-Zone High Gray-level Em-phasis; LZLGE: Long-Zone Low Gray-level Emphasis; LZHGE: Long-Zone High Gray-level Emphasis; ZLNU: Zone Length Non-Uniformity.Extraction of RF by LIFEx is only possible for VOI of at least 64 voxels, therefore 16 patients were excluded from the study because the volume of the TIs uptake was below this limit. As a consequence, the final number of patients included in the study was 221.

**Table 2:** Characteristics of the 221 patients included in the study.

Characteristic	N. (%)
Age, mean ± SD (range)	66 ± 14 (16–88)
Sex	
Male	72 (33%)
Female	149 (67%)
Thyroid Lobe	
Right	123 (56%)
Left	87 (39%)
Isthmus	11 (5%)
Ultrasound diameter (mm), mean ± SD (range)	17 ± 12 (5–75)
Final Diagnosis	
Benign	150 (68%)
Malign	71 (32%)
Cytology (N. = 118)	
TIR2	35 (30%)
TIR3a	24 (20%)
TIR3b	30 (25%)
TIR4	13 (11%)
TIR5	16 (14%)
Histology (N. = 71)	
Anaplastic carcinoma	3 (4%)
Follicular carcinoma	7 (10%)
Papillary carcinoma	61 (86%)
PET/CT Scanner	
Scanner 1 (Discovery 690)	128 (58%)
Scanner 2 (Discovery STE)	93 (42%)
Semiquantitative PET/CT parameters	
SUVmax, mean ± SD (range)	7.9 ± 8 (1.3–56.7)
SUVmean, mean ± SD (range)	4.3 ± 4 (1.0–37.1)
SUVlbm, mean ± SD (range)	5.8 ± 6 (1.0–41.3)
SUVbsa, mean ± SD (range)	2.0 ± 2 (0.4–12.6)
MTV, mean ± SD (range)	9.2 ± 18 (0.4–198.0)
TLG, mean ± SD (range)	35.0 ± 75 (1.9–722.4)

N.: number, SD: standard deviation, mm: millimeters, SUVmax: standardized uptake value body weight max, SUVmean: standardized uptake value body mean, SUVlbm: standardized uptake value lean body mass, SUVbsa: standardized uptake value body surface area, MTV: metabolic tumor volume, TLG: total lesion glicolysis.

**Table 3:** Comparison of clinical parameters, semiquantitative PET/CT parameters and radiomics features between the two scanners.

Parameters	*p*-Value
Clinical	
Age	0.787
Sex	0.522
Diameters at ultrasound	0.446
Semiquantitative PET/CT parameters	
SUVmax	0.046
SUVmean	0.118
SUVlbm	0.119
SUVbsa	0.076
MTV	0.595
TLG	0.869
Radiomics features	
Histo skewness	0.193
Histo kurtosis	0.924
Histo excess kurtosis	0.924
Histo entropy_log10	0.023
Histo entropy_log2	0.024
Histo energy	0.017
Shape volume_mL	0.211
Shape volume_vx	0.560
Shape sphericity	0.088
Shape compacity	0.518
GLCM homogeneity	0.104
GLCM energy	0.638
GLCM contrast	0.132
GLCM correlation	0.889
GLCM entropy_log10	0.319
GLCM entropy_log2	0.315
GLCM dissimilarity	0.145
GLRLM SRE	0.123
GLRLM LRE	0.113
GLRLM LGRE	0.026
GLRLM HGRE	0.069
GLRLM SRLGE	0.036
GLRLM SRHGE	0.069
GLRLM LRLGE	0.098
GLRLM LRHGE	0.135
GLRLM GLNU	0.260
GLRLM RLNU	0.962
GLRLM RP	0.126
NGLDM coarseness	0.471
NGLDM contrast	0.476
NGLDM busyness	0.006
GLZLM SZE	0.017
GLZLM LZE	0.168
GLZLM LGZE	0.053
GLZLM HGZE	0.086
GLZLM SZLGE	0.069
GLZLM SZHGE	0.041
GLZLM LZLGE	0.102
GLZLM LZHGE	0.561
GLZLM GLNU	0.366
GLZLM ZLNU	0.026
GLZLM ZP	0.093

**Table 4:** Univariate analysis for semiquantitative PET/CT parameters and for radiomics features for the single scanner and for both scanners considered together. Only values with AUC > 0.6 and *p*-value < 0.05 are reported.

	Mean AUC			Mean *p*-Value		
Parameters	Scanner 1	Scanner 2	Scanner 1 + 2	Scanner 1	Scanner 2	Scanner 1 + 2
SUVmax	0.762	0.679	0.748	<0.01	0.02	<0.01
SUVmean	0.724	0.675	0.748	<0.01	<0.01	<0.01
SUVlbm	0.757	0.685	0.748	<0.01	0.01	<0.01
SUVbsa	0.756	0.689	0.742	<0.01	0.01	<0.01
Histo entropy_log10	0.709	0.674	0.724	<0.01	<0.01	<0.01
Histo entropy_log2	0.705	0.674	0.724	<0.01	<0.01	<0.01
GLCM entropy_log10	0.713	0.664	0.702	0.02	0.03	<0.01
GLCM entropy_log2	0.712	0.664	0.703	0.02	0.03	<0.01
GLCM dissimilarity	0.719	0.682	0.727	0.01	<0.01	<0.01
GLRLM HGRE	0.731	0.693	0.741	0.03	0.03	<0.01
GLRLM SRHGE	0.739	0.682	0.744	0.02	0.02	<0.01
GLRLM LRLGE	0.707	0.653	0.715	0.01	0.01	<0.01
GLZLM SZE	0.734	0.671	0.693	<0.01	<0.01	0.01
GLZLM HGZE	0.740	0.668	0.740	0.02	0.03	<0.01
GLZLM SZHGE	0.758	0.693	0.733	0.02	0.03	<0.01
GLZLM ZP	0.692	0.669	0.699	<0.01	0.01	<0.01
Variables with good performances only at Scanner 1 + 2 analysis						
GLCM contrast	0.733			0.01		
GLZLM ZLNU	0.729			0.04		
GLRLM LRLGE	0.715			<0.01		
GLZLM LGZE	0.706			<0.01		
GLRLM LGRE	0.703			<0.01		
GLCM homogeneity	0.702			<0.01		
GLRLM SRLGE	0.687			<0.01		
NGLDM busyness	0.684			0.01		
GLRLM RP	0.660			0.04		
GLZLM SZLGE	0.651			<0.01		

AUC: area under the curve.

**Table 5:** Bivariate analysis for clinical, semiquantitative PET/CT parameters and radiomics features for the single scanner and for both scanners considered together. For each analysis, only the couples with best performances are reported.

Covariate 1	Covariate 2	Mean *p*-Value 1	Mean *p*-Value 2	Mean AUC
Scanner 1				
GLZLM GLNU	MTV	<0.01	0.01	0.779
GLRLM RLNU	MTV	0.02	0.03	0.776
GLCM energy	GLCM entropy_log2	0.04	<0.01	0.771
GLCM energy	GLCM entropy_log10	0.04	<0.01	0.771
GLCM entropy_log2	GLRLM HGRE	0.01	0.03	0.763
GLCM entropy_log10	GLZLM HGZE	0.02	0.02	0.762
GLCM entropy_log10	GLRLM HGRE	0.01	0.03	0.761
GLCM entropy_log2	GLZLM HGZE	0.02	0.02	0.760
GLCM entropy_log10	GLZLM SZHGE	0.01	0.02	0.760
GLCM entropy_log2	GLZLM SZHGE	0.01	0.02	0.759
GLRLM RP	GLZLM SZHGE	0.04	0.02	0.751
GLRLM HGRE	GLRLM RP	0.02	0.03	0.745
MTV	TLG	<0.01	0.01	0.741
GLRLM SRE	GLZLM HGZE	0.03	0.01	0.740
NGLDM coarseness	NGLDM busyness	<0.01	0.01	0.738
Shape volume_mL	GLRLM GLNU	0.03	0.01	0.736
GLRLM GLNU	NGLDM coarseness	0.03	<0.01	0.734
GLRLM SRE	GLZLM SZHGE	0.03	0.02	0.732
GLRLM SRE	GLRLM HGRE	0.03	0.02	0.730
GLRLM LRLGE	NGLDM coarseness	<0.01	0.04	0.730
Shape volume_vx	GLRLM GLNU	0.02	0.02	0.723
GLCM entropy_log10	GLZLM SZHGE	0.04	<0.01	0.713
Shape compacity	GLZLM GLNU	0.01	<0.01	0.707
Shape volume_mL	MTV	0.02	0.02	0.693
Ultrasound dimension	MTV	0.01	0.02	0.691
GLCM correlation	NGLDM coarseness	<0.01	<0.01	0.690
Shape compacity	NGLDM coarseness	0.03	0.01	0.680
Ultrasound dimension	GLRLM GLNU	0.01	0.01	0.677
Scanner 2				
GLRLM SRE	SUVmean	0.04	0.01	0.712
GLCM entropy_log10	SUVbsa	0.05	0.01	0.697
GLCM entropy_log2	SUVbsa	0.05	0.02	0.696
GLCM entropy_log2	GLZLM SZHGE	0.03	0.02	0.689
GLCM entropy_log10	GLZLM SZHGE	0.03	0.02	0.689
GLRLM RP	SUVmean	0.05	0.02	0.686
GLCM entropy_log2	GLZLM HGZE	0.05	0.02	0.682
GLCM entropy_log10	GLZLM HGZE	0.05	0.02	0.680
GLCM entropy_log10	GLRLM HGRE	0.04	0.02	0.679
GLCM energy	GLRLM LRHGE	0.01	0.02	0.679
GLCM entropy_log2	GLRLM HGRE	0.04	0.02	0.679
NGLDM coarseness	GLZLM ZP	0.03	<0.01	0.677
Histo energy	GLRLM HGRE	0.04	0.03	0.676
GLCM homogeneity	NGLDM coarseness	<0.01	0.06	0.675
GLCM contrast	GLCM entropy_log10	0.04	0.04	0.673
GLCM contrast	GLCM entropy_log2	0.04	0.04	0.673
Histo energy	GLZLM SZHGE	0.04	0.03	0.669
GLRLM SRE	GLRLM HGRE	0.04	0.03	0.669
GLRLM SRE	NGLDM coarseness	0.01	0.05	0.668
GLRLM LRE	SUVmean	0.04	0.01	0.668
NGLDM coarseness	NGLDM busyness	0.02	0.02	0.666
GLZLM GLNU	MTV	0.02	0.02	0.663
GLCM energy	GLZLM SZHGE	0.06	<0.01	0.660
GLCM energy	GLRLM HGRE	0.06	<0.01	0.659
GLRLM RP	NGLDM coarseness	0.01	0.05	0.657
GLRLM RLNU	MTV	0.01	0.01	0.650
NGLDM coarseness	MTV	0.04	0.03	0.627
Scanner 1 + 2				
GLCM entropy_log2	GLZLM SZHGE	<0.01	<0.01	0.769
GLCM entropy_log10	GLRLM HGRE	<0.01	<0.01	0.769
GLCM entropy_log10	GLZLM SZHGE	<0.01	<0.01	0.769
GLCM entropy_log10	GLZLM HGZE	<0.01	<0.01	0.769
GLCM entropy_log2	GLRLM HGRE	<0.01	<0.01	0.768
GLCM entropy_log2	GLZLM HGZE	<0.01	<0.01	0.768
GLRLM SRE	SUVmean	<0.01	<0.01	0.763
GLRLM GLNU	NGLDM Coarseness	<0.01	<0.01	0.756
GLCM homogeneity	GLRLM HGRE	<0.01	<0.01	0.749
GLCM homogeneity	GLZLM HGZE	<0.01	<0.01	0.749
Histo energy	GLRLM HGRE	<0.01	<0.01	0.749
Histo energyUniformity	GLZLM SZHGE	<0.01	<0.01	0.748
GLCM homogeneity	GLZLM SZHGE	<0.01	<0.01	0.748
NGLDM coarseness	NGLDM busyness	<0.01	<0.01	0.746
GLRLM SRE	GLRLM HGRE	<0.01	<0.01	0.742
GLRLM RP	GLZLM HGZE	<0.01	<0.01	0.742
GLRLM SRE	GLZLM HGZE	<0.01	<0.01	0.742
GLRLM HGRE	GLRLM RP	<0.01	<0.01	0.742
NGLDM coarseness	GLZLM ZP	<0.01	<0.01	0.741
GLZLM GLNU	MTV	<0.01	<0.01	0.738
GLRLM SRE	GLZLM SZHGE	<0.01	<0.01	0.738
GLRLM RP	GLZLM SZHGE	<0.01	<0.01	0.737
GLRLM LRE	GLRLM LRHGE	<0.01	<0.01	0.737
GLRLM RLNU	MTV	<0.01	<0.01	0.730
Histo energy	GLCM energy	<0.01	<0.01	0.717
Shape compacity	NGLDM coarseness	<0.01	<0.01	0.681
GLCM correlation	NGLDM coarseness	<0.01	<0.01	0.654
Shape compacity	GLZLM GLNU	<0.01	<0.01	0.640

AUC: area under the curve.

## Data Availability

Data are not public, but are present in our institution.
